# Student’s Guide to Diseases of the Eye

**Published:** 1883-05

**Authors:** 


**Student's Guide to Diseases of the Eye.** By EDWARD NETTLESHIP, F, R. C. S., Ophthalmic Surgeon to St. Thomas Hospital. Second American from the second revised and enlarged English edition, with a chapter on examination for color perception, by William Thompson, M. D., Professor of Ophthalmology at Jefferson Medical College. Philadelphia : Henry C. Lea's Son & Co. 1883.

This work is essentially a students manual of ophthalmology, and the favor with which it has been received shows its real value in this special department to which it is devoted, and the appreciation of the profession of its intrinsic merits. Dr. Thompson has added a chapter on color-blindness, in which subject his extensive investigations are widely known ; with this valuable addition the book becomes the most valuable guide to the study of diseases of the eye yet published. We commend it to the notice of students of medicine, and to such practitioners as desire a condensed treatise on a class of diseases which are frequently met with in daily practice.

